# The piRNA size matters

**DOI:** 10.1093/nsr/nwad297

**Published:** 2023-11-22

**Authors:** Jingwen Liu, Falong Lu

**Affiliations:** State Key Laboratory of Molecular Developmental Biology, Institute of Genetics and Developmental Biology, Chinese Academy of Sciences, China; University of Chinese Academy of Sciences, China; State Key Laboratory of Molecular Developmental Biology, Institute of Genetics and Developmental Biology, Chinese Academy of Sciences, China; University of Chinese Academy of Sciences, China

Small RNAs, including small interfering RNAs (siRNAs), microRNAs (miRNAs) and PIWI-interacting RNAs (piRNAs), serve as pivotal agents in the post-transcriptional regulation of gene expression [1–3]. Among these, piRNAs take on multifaceted roles, being indispensable for repressing transposable elements to safeguard genome stability within the germline and concurrently overseeing the post-transcriptional modulation of protein-coding mRNAs with regard to their stability and translational efficiency [3–7]. A unique characteristic setting the piRNA class apart is their extended length, typically spanning 24–33 nucleotides (nt)—a marked contrast to the shorter sizes exhibited by siRNAs or miRNAs (∼21 nt). piRNAs are loaded onto effector Argonaute proteins that form a specific clade called the PIWI-clade Argonaute family of proteins to execute their functions. However, it remains largely unknown why PIWI-clade proteins bind piRNAs of different sizes and whether the size is functionally significant. In a recent study, Wang *et al.* elucidated the function of a distinctive insertion module residing within PIWI proteins, which helps to define the length of piRNAs [8]. Notably, this new finding establishes the pivotal role of piRNA length in the translational regulation orchestrated by the PIWI/piRNA complex that stands as crucial for maintaining male fertility in both mice and humans. This revelation underscores a fundamental role of the piRNA size in specifying its roles in mammalian germ cells.

During the piRNA production, a long single-stranded piRNA intermediate is loaded onto the PIWI proteins with its 5′ end anchored within the protein, while its 3′ end is formed by endonucleolytic cleavage followed by exonucleolytic trimming of extra nucleotides not protected by PIWI binding [9,10]. PIWI-clade proteins contain a PIWI-specific Insertion (PIWI-Ins), which may facilitate the binding of longer piRNAs as revealed by the crystal structure of silkworm PIWI (Siwi) protein [11]. In their recent study, Wang *et al.* performed an elegant study into the significance of piRNA size through manipulation of the PIWI-Ins

in mice. To commence, the authors unveiled the conservation of a PIWI-Ins module across mammalian PIWI-clade proteins, being able to accommodate longer piRNAs through structural prediction using AlphaFold. Deletion of the 9-amino acid PIWI-Ins within mouse PIWI (MIWI) results in the production of shortened piRNAs, whereas the substitution of the 9-amino acid PIWI-Ins with a lengthier 27-amino acid variant from planarian PIWI protein results in the generation of extended piRNAs (Fig. 1), establishing the decisive role played by the PIWI-Ins module in determining the length of loaded piRNAs.

**Figure 1.**
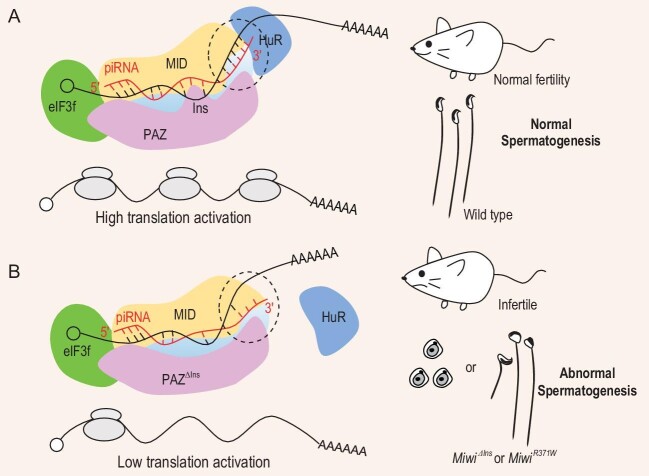
Schematic illustrations of the role of the PIWI-Ins/piRNA length in translational activation and spermatogenesis. (A) PIWI-specific Insertion (PIWI-Ins) accommodates longer piRNAs that extend the piRNA 3′ end target complementarity (indicated by the black dotted circle). This piRNA 3′ end base-pairing promotes target mRNA translational activation by recruiting the HuR into the MIWI/eIF3f/HuR super-complex, which is essential for spermatogenesis in both mice and humans. (B) PIWI-Ins deletion leads to short piRNA, which reduces piRNA 3′ end target complementarity (indicated by the black dotted circle) and fails to recruit HuR to assemble the MIWI/eIF3f/HuR super-complex, which leads to low translation activation of target mRNA. The PIWI-Ins mutation leads to abnormal sperm and infertility in both mice and humans.

Remarkably, the deletion of the PIWI-Ins module in *Miwi* has led to complete male sterility in mice, characterized by the absence of mature spermatozoa within the epididymis (Fig. 1). When compared with the more severe phenotype observed in *Miwi*-null mice [12], the impact of PIWI-Ins deletion appears comparatively moderated. This discernible contrast implies that the PIWI-Ins module plays a pivotal role in facilitating the functionality of MIWI during the post-meiotic phase of male germ cell development.

In-depth molecular analysis reveals that, while there is a similar level of piRNA expression and MIWI loading capacity in PIWI-Ins module-deleted *Miwi* mutant mice, piRNAs associated with PIWI-Ins-deleted MIWI protein are shorter than those associated with wild type MIWI. This *in vivo* molecular observation is similar to the biochemical observation that PIWI-Ins helps to define the length of the piRNAs loaded onto MIWI. Surprisingly, the shorter piRNA and PIWI-Ins-deleted MIWI maintain their ability to effectively repress their transposable element targets, known as the fundamental function of the PIWI/piRNA pathway. In accordance with this observation, the transcriptome is also not significantly changed upon deletion of PIWI-Ins of MIWI. Very interestingly, MIWI/piRNA-mediated translational activation in round spermatids is impaired upon PIWI-Ins deletion in MIWI, along with mild defects in the cleavage of some specific target mRNAs of piRNAs.

Mechanistically, base-pairing between the 3′ end of piRNAs and their target mRNAs is positively associated with the role of piRNAs in translational activation of their mRNA targets, as evidenced by manipulating the piRNA 3′ base-pairing or the 3′ end length. This piRNA 3′ base-pairing with its target mRNA greatly contributes to translational activation of its target mRNA. At the biochemical level, the piRNA 3′ end target complementarity is required for the recruitment of the HuR subunit into the MIWI/eIF3f/HuR super-complex (Fig. 1). In support of this mechanism, standard piRNA force-loaded into the PIWI-Ins-deleted MIWI through exogenous transfection culminates in the normal formation of the MIWI/eIF3f/HuR super-complex. Interestingly, increased complementarity at the 3′ end of a piRNA to its normally repressed target mRNA can enhance HuR binding, which can switch the role of piRNA from a repressor to a potent activator.

Mutations within the human *PIWIL1* (*HIWI*) gene, the ortholog of *Miwi*, have been implicated in patients suffering from azoospermia [13,14]. Importantly, researchers have identified a patient with compound heterozygous mutations in *HIWI* (*HIWI^R439X/R370W^*) among patients grappling with male infertility. The p.R439X mutation causes HIWI truncation, which is likely a loss-of-function allele. Interestingly, the p.R370W mutation is within the PIWI-Ins module. This discovery is significant given that its counterpart, the MIWI p.R371W mutation (corresponding to the p.R370W mutation in HIWI), causes subfertility in male mice (Fig. 1). This mouse model demonstrates a marked increase in abnormal sperm exhibiting diminished fertilization capability, rendering the mice subfertile. Moreover, these animals encounter a premature loss of fertility, unfolding critical insights into the consequences of the analogous mutation in the *Miwi* ortholog, *HIWI*.

Collectively, this work by Wang *et al.* has unveiled an unforeseen finding that the length of piRNA and its complementarity with its target mRNA at its 3′ end is a critical regulatory factor of piRNA function in translational activation during spermatogenesis. This study provides elegant mechanistic understanding of this regulation, elucidating the significance of a PIWI-specific Insertion within PIWI-clade proteins, representing a breakthrough in understanding the function and regulation of piRNA length. Importantly, this new discovery also carries profound implications in the diagnosis and therapeutic strategies for male infertility in patients.

## References

[1] Bartel DP . Cell 2018; 173: 20–51. 10.1016/j.cell.2018.03.006 29570994 PMC6091663

[2] Chen X , Rechavi O. Nat Rev Mol Cell Biol 2022; 23: 185–203. 10.1038/s41580-021-00425-y 34707241 PMC9208737

[3] Wang X , Ramat A, Simonelig M et al. Nat Rev Mol Cell Biol 2023; 24: 123–41. 10.1038/s41580-022-00528-0 36104626

[4] Dai P , Wang X, Gou LT et al. Cell 2019; 179: 1566–81. 10.1016/j.cell.2019.11.022 31835033 PMC8139323

[5] Dai P , Wang X, Liu MF. Sci China Life Sci 2020; 63: 447–9. 10.1007/s11427-020-1632-5 31974861

[6] Gou LT , Dai P, Yang JH et al. Cell Res 2015; 25: 266. 10.1038/cr.2015.14 25645811 PMC4650571

[7] Wang X , Gou LT, Liu MF. Biol Reprod 2022; 107: 101–8. 10.1093/biolre/ioac073 35403682

[8] Wang X , Lin DH, Yan Y et al. Sci China Life Sci 2023; 66: 1459–81. 10.1007/s11427-023-2390-5 37335463

[9] Han BW , Wang W, Li CJ et al. Science 2015; 348: 817–21. 10.1126/science.aaa1264 25977554 PMC4545291

[10] Mohn F , Handler D, Brennecke J. Science 2015; 348: 812–7. 10.1126/science.aaa1039 25977553 PMC4988486

[11] Matsumoto N , Nishimasu H, Sakakibara K et al. Cell 2016; 167: 484–97. 10.1016/j.cell.2016.09.002 27693359

[12] Deng W , Lin H. Dev Cell 2002; 2: 819–30. 10.1016/S1534-5807(02)00165-X 12062093

[13] Gou LT , Kang JY, Dai P et al. Cell 2017; 169: 1090–104. 10.1016/j.cell.2017.04.034 28552346 PMC5985145

[14] Hasuwa H , Ishino K, Siomi H. Sci China Life Sci 2018; 61: 348–50. 10.1007/s11427-017-9149-0 28801861

